# Advanced differential evolution for gender-aware English speech emotion recognition

**DOI:** 10.1038/s41598-024-68864-z

**Published:** 2024-07-31

**Authors:** Liya Yue, Pei Hu, Jiulong Zhu

**Affiliations:** 1 Fanli Business School, Nanyang Institute of Technology, Nanyang, 473004 China; 2 School of Computer and Software, Nanyang Institute of Technology, Nanyang, 473004 China

**Keywords:** Emotion recognition, Gender, Differential evolution, Computer science, Data mining

## Abstract

Speech emotion recognition (SER) technology involves feature extraction and prediction models. However, recognition efficiency tends to decrease because of gender differences and the large number of extracted features. Consequently, this paper introduces a SER system based on gender. First, gender and emotion features are extracted from speech signals to develop gender recognition and emotion classification models. Second, according to gender differences, distinct emotion recognition models are established for male and female speakers. The gender of speakers is determined before executing the corresponding emotion model. Third, the accuracy of these emotion models is enhanced by utilizing an advanced differential evolution algorithm (ADE) to select optimal features. ADE incorporates new difference vectors, mutation operators, and position learning, which effectively balance global and local searches. A new position repairing method is proposed to address gender differences. Finally, experiments on four English datasets demonstrate that ADE is superior to comparison algorithms in recognition accuracy, recall, precision, F1-score, the number of used features and execution time. The findings highlight the significance of gender in refining emotion models, while mel-frequency cepstral coefficients are important factors in gender differences.

## Introduction

Voice signals convey emotions, beyond just words and meanings. In addition to human facial expressions, speech is another promising way to recognize human emotions^1,2^. Human oral communication intricately connects to linguistic information (speech content) and paralinguistic information (such as tone, emotional state, and gestures)^3,4^. The emotional state of a person is instrumental in enriching communication.

Speech emotion recognition (SER) does not consider semantic content but instead focuses on identifying speakers’ emotional states such as happiness, sadness, fear, etc^5,6^. Without explicit inquiry, human-computer interaction heavily depends on the ability to recognize and interpret speakers’ emotions^7,8^. In the medical field, effective emotion recognition can improve speech intelligibility for individuals with speech impairments and help people better understand the conveyed information. In education, analyzing the online learning experiences of students can assess their emotional states and enhance teaching quality. In criminal investigations, automatic speech recognition can reveal the true emotional states of suspects and detect their attempts to conceal emotions^9^.

Acoustic features employed in SER include mel-frequency cepstral coefficient (MFCC), linear prediction coefficient (LPC), zero crossing rate, logarithmic frequency power coefficient (LFPC), and high-level prosodic features^10,11^. This results in obtaining high-dimensional emotional features. Feature selection improves the overall performance of SER, and reduces computational complexity^12–14^.

While differential evolution (DE) is an effective metaheuristic algorithm, its population diversity and convergence speed decrease drastically during evolution^15,16^. Zeng proposed a new selection operator to enhance the performance of DE^17^. When an individual is not in a stagnant state, the proposed operator is similar to the classical selection, meaning that it selects the best vector from trial and parent vectors to survive to the next generation. When an individual is in a stagnant state, the other three candidate vectors have a chance of surviving to the next generation. The first and second candidates are the best and second-best vectors among all discarded trial parents, while the third candidate is randomly selected from all successfully updated solutions. This proposed selection operator improves the ability of DE to escape local optima. Gupta and Su introduced a new mutation strategy based on guiding individuals to improve convergence and diversity^18^. The basic vector is the center for guiding individuals, while the difference vector is used to locate one of the available best individuals. The control parameter is adjusted to provide an appropriate transition from exploration to exploitation, and to make use of knowledge from recent successful evolutionary history. To ensure that DE achieves fast convergence, Lin et al. designed a framework that takes the advantages of different mutation strategies^19^. Firstly, an improved mean individual mutation strategy is integrated into the DE algorithm to enhance global convergence. Secondly, the DE/current-to-rand/1 strategy is used to improve diversity and generate disturbances to prevent the algorithm from getting stuck in local optimum. Lastly, a perturbation strategy is proposed to assist the population in escaping from local traps and improve its exploration ability. To address issues like local optimal stagnation and numerical instability in large-scale feature selection, Wang et al. claimed a new DE^20^. First, they adopt a multi-population strategy to enhance population diversity. Then, a new adaptive mechanism is employed to select multiple policies from a policy pool to acquire various information from historical solutions. Finally, a weighted model is designed to identify important features, which enables the model to generate the most appropriate feature selection.

Based on the above analysis, the improvement of DE often involves mutation operators and parameter control. It is necessary to consider that DE is limited by positions when performing feature selection, and we design novel operators. The main contributions of this paper are summarized as follows:

1. We present a speech emotion recognition system based on gender-aware to address the differences between male and female voices.
2. We propose a DE algorithm for feature selection in emotion recognition.
3. We verify the superiority of the proposed algorithm (ADE) on English speech emotion datasets using multiple metrics, and ADE identifies features influencing emotion and gender.

The organization of this paper is as follows. “Related works” section presents the related works of speech emotion recognition. “Materials and methods” section describes the proposed system. “Experimental results and discussions” section presents the experimental results with discussions, while “Conclusions” section offers the conclusions.

## Related works

Speech signals play an important role in human-computer interaction, and serve as the primary input source for various applications, such as speech recognition, speech emotion recognition, and gender recognition^21,22^. Recently, it has emerged as a prominent research field to automatically extract speakers’ gender and emotional state from speech signals. Various approaches have been explored by researchers to enhance the accuracy of emotion recognition.

Emotion recognition is becoming more popular due to its various applications, but it faces challenges arising from factors like corpus differences, speaker gender, and expression domains (spoken or sung). Zhang et al. studied the impact of these factors on the generalizability of emotion recognition systems across multiple corpora^23^. They used factors to define a multi-task learning method which incorporates variability due to the corpus. In the domain of speech, gender and corpus have equal influence. Sun presented a new SER algorithm that doesn’t rely on acoustic features and incorporates speakers’ gender information^24^. The goal is to obtain rich information from raw speech data without any human intervention. Unlike conventional speech emotion recognition systems that demand manual selection of acoustic features, the approach employs deep learning algorithms to automatically extract essential information from original speech signals. It prevents the lack of emotional information that cannot be mathematically modeled as acoustic features. Velichko et al. introduced a hierarchical framework for intricate paralinguistic speech analysis^25^, including gender, emotion, and deception recognition. The foundation of this framework is the study of the interrelationships between different paralinguistic phenomena. It employs gender information to predict emotional states and uses the results of emotion recognition to predict the authenticity of speech. Aggarwal et al. used naive Bayesian and support vector machine (SVM) to recognize emotions and gender^26^, and utilized four speech features: shimmer, jitter, energy, and pitch. The findings suggest that SVM exhibits higher accuracy in gender and emotion recognition compared to naive Bayesian.

Mishra et al. developed a two-stage emotion recognition model for gender-distinguished speech that utilizes MFCCs and a convolutional neural network (CNN)^27^. Gender-independent emotion recognizers are less effective than gender-dependent emotion recognizers due to the acoustic differences between male and female speakers. The results show that systems with gender recognition have a significant impact on performance. Notably, the performance is enhanced by employing a global average pool at the end of the CNN classifier. Latif et al. brought a multi-task framework that uses gender-speaker recognition as supplementary tools for emotion classification^28^. To maximize the effectiveness of multi-task learning, adversarial autoencoders (AAE) are integrated into the framework, which have strong learning and discriminative feature abilities. Furthermore, the combination of unsupervised AAE and a supervised classification network achieves the semi-supervised learning that improves the generality of the framework and the overall performance of SER.

Garain et al. converted input speech signals into spectral images^29^, and extracted a set of common features for gender, speaker, and emotion recognition tasks. The mayfly algorithm chooses features with minimal redundancy and maximum relevance (mRMR). Due to the challenging issue of determining the number of units per layer in MLP, the golden ratio is utilized to complete this task.

Yao et al. developed a framework that integrates three different classifiers: deep neural network (DNN), convolutional neural network (CNN), and recurrent neural network (RNN)^30^. This framework is used to classify and recognize four emotions: anger, happiness, neutrality, and sadness. To address feature confusion issues that complicate accurate emotion classification, Liu et al. employed a cascaded attention network for SER^31^. This network selectively discovers target emotional regions from MFCC features using spatiotemporal attention and employs a joint loss function to distinguish highly similar emotion embeddings to improve overall performance. Deep learning models are suitable for processing large, complex, and high-dimensional data and can automatically extract advanced features from the data. Evolutionary algorithms, with their powerful global search capabilities and independence from gradient information, are employed to optimize complex, discontinuous problems. Deep learning methods are widely utilized in recognizing emotions in images and texts and perform exceptionally well. Evolutionary algorithms are suitable for optimizing model structures, feature selection, and solving complex optimization problems.

DE is a simple and powerful metaheuristic algorithm for solving global optimization problems, and it has been widely applied in various fields because of its concise structure and robust search capabilities. We use DE to filter emotional features and incorporate them into prediction models. We also introduce a new method for repairing positions that can address the issue of gender differences in speech.

## Materials and methods

**Figure 1:**
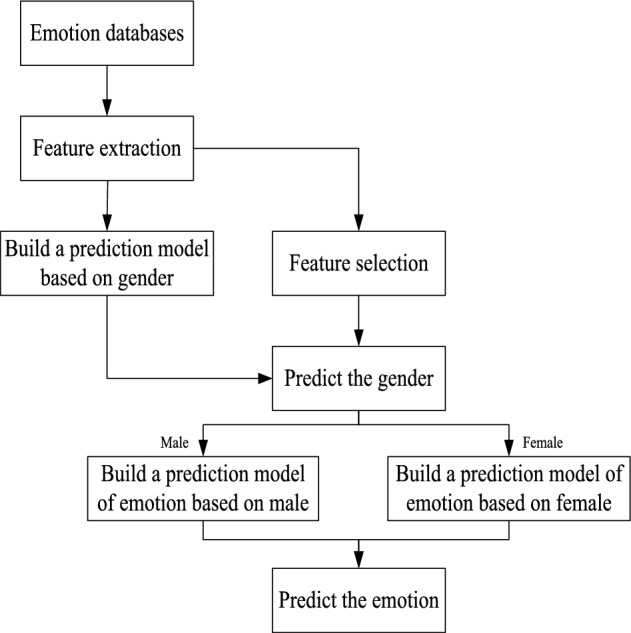
The flowchart of the proposed system.

The proposed system consists of emotional databases, feature extraction, and feature selection based on DE, and Fig. 1 presents the flowchart. This system builds a gender prediction model by extracting features from emotion databases. When using DE to implement feature selection on the extracted emotion features, it first predicts the gender of speakers, and then builds a gender-based emotional model to achieve accurate prediction.

### Emotional databases

This study employs CREMA-D^32^, EmergencyCalls^33^, IEMOCAP-S1^34^, and RAVDESS^35^ to implement emotion recognition based on gender.

1. CREMA-D CREMA-D is a dataset of 7,442 original clips from 91 actors. These clips are from 48 male and 43 female actors between the ages of 20 and 74 coming from a variety of races. The actors speak from a selection of 12 sentences, while sentences are presented with one of six different emotions: Angry, Fearful, Disgust, Neutral, Happy, and Sad.
2. EmergencyCalls The database is from Kaggle. 18 speakers (9 males and 9 females) are asked four sentences to record their audios in four emotions: Angry, Drunk, Painful and Stressful.
3. IEMOCAP-S1 Unlike many emotion databases that involve single speakers, the characteristic of IEMOCAP is the participation of multiple persons in various scenarios. The IEMOCAP-S1 database is a session of IEMOCAP, and it includes many emotions, such as Neutral, Happy, Sad, Angry, Surprised, Fearful, Disgust, Frustrated, Exciting, and Other.
4. RAVDESS In RAVDESS, there are 24 professional actors (12 female and 12 male) who speak two lexically-matched sentences with a neutral North American accent. Speech emotions include Fearful, Calm, Surprised, Happy, Angry, Sad, and Disgust expressions.

### Feature extraction

We employ pitch features to identify gender and the OpenSmile toolkit^36^ to obtain acoustic features for recognizing emotions. Table 1 describes their details in which gender contains 11 features and emotion involves 113 values. A total of 124 features are utilized for emotion recognition.

**Table 1:** Summary of the extracted features.

Category	Description	Features
Gender	Pitch	Maximum, minimum, median, mean, variance and derivatives
	Spurt	Length
Emotion	PCM Loudness	Position- max./min.
	MFCC [0-14]	Arith. mean, std. deviation
	F0 by Sub-Harmonic Sum	Lin. regression error Q/A-
	F0 Envelope Quartile	Quartile- 1/2/3
	Log Mel Freq. Band [0-7]	Skewness, kurtosis
	LSP Frequency [0-7]	Lin. regression coeff.- 1/2
	Shimmer Local	Up-level time- 75/90
	Voicing Probability	Quartile range- 2-1/3-2/3-1
	Jitter Local	Percentile 1/99
	Jitter DDP	Percentile range 99-1

### Advanced differential evolution

**Figure 2:**
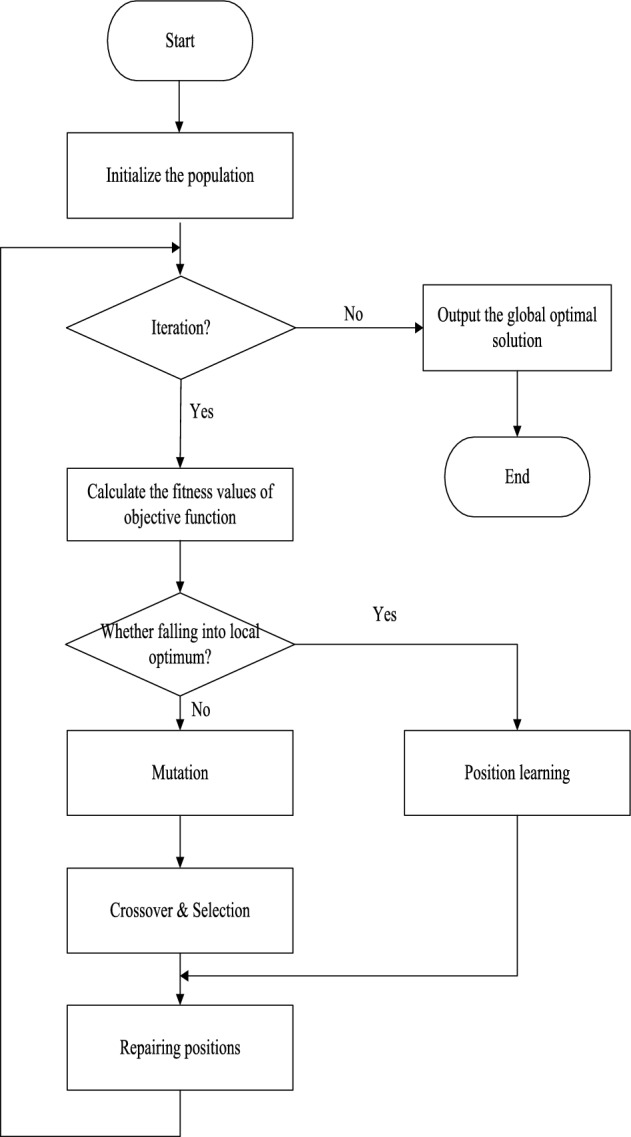
The flowchart of ADE.

Difference vectors and the scale factor affect the performance of DE. To improve its diversity, we introduce a novel selection of basis and difference vectors during the mutation process. The scale factor, based on differentiation, accelerates the convergence speed of the population. Additionally, a new position learning strategy is proposed to prevent stagnation in local optima throughout the evolution process. The flowchart of ADE is illustrated in Fig. 2.

#### Mutation

Individuals in DE update their positions through mutation and crossover, as depicted in Eqs. (1) and (2). If the objective function value of a new individual surpasses that of the existing individual, the latter is substituted. Otherwise, the original individual is retained.

1 $$\begin{aligned} m_{i}(t+1) = X_{r_1}(t) + F * (X_{r_2}(t) - X_{r_3}(t)) \end{aligned}$$

2 $$\begin{aligned} X_i^j(t+1)=\left\{ \begin{array}{l} m_{i}^j(t+1) \qquad if(rand(j) \le PCR) \quad or \quad j = randi() \\ X_{i}^j(t) \qquad \qquad else \end{array} \right. \end{aligned}\qquad$$

where $X_{i}^j(t)$ represents the position of individual *i* in the j-th dimension of the t-th iteration. *m* is a trail value, and *F* means the scale factor. *PCR* is a crossover probability.

The effectiveness of mutation is strongly impacted by the choice of basis and parent vectors. Therefore, their correct selection is crucial to balance diversity and convergence and to supply promising search guidance. We first rank the fitness values of the population, and then update the positions of only the worst half of individuals. The other half of individuals serve as guidance vectors; that is, $r_1$, $r_2$ and $r_3$ in Eq. (1) come from this part.

Individuals with high objective function values bring useful insights into promising regions, while those with lower fitness values can identify less promising areas. Superior individuals guide exploration, whereas less ones indicate areas to avoid. In our approach, outstanding individuals act as attractors, and lower-performing individuals act as repellers. If the fitness value of $r_2$ is inferior to $r_3$, we multiply *F* by -1, which can ensure that the population quickly moves closer to the optimal solution.

New basis and difference vectors improve convergence, and a differential-based scale factor is proposed to balance exploration and exploitation during the optimization process.

3 $$\begin{aligned} F_i(t+1) = \dfrac{abs(f_i(t) - f_i(t-1))}{t-(t-1)} \end{aligned}$$

where *f* represents the objective function value, and $F_i(1)$ is set to the default value of DE. Due to the fast convergence of the algorithm, it is easy to cause *F* to become too small, and *F* cannot affect individuals’ update. Consequently, the objective function values of the updated individuals are sorted from good to bad, and the value of *F* is normalized to [0.2, 0.8]. To increase search diversity, a mutation within the range of [-0.1, 0.1] is randomly performed for each dimension.

#### Position learning

In the early stages of ADE, individuals are distributed throughout the search space, and global search is an efficient method for gathering evolutionary information. With the search of the population, individuals tend to cluster in several excellent areas, and it is necessary to fully consider both global and local searches. In the final stages of the algorithm, a slower convergence is caused by individual differences, and search capabilities tend to be localized. If the population does not update the global optimal solution for ten cycles, they will be compelled to leave the current search area.

4 $$\begin{aligned} X_i^j(t+1)=\left\{ \begin{array}{l} 1/2 + (1/2 - X_i^j(t)) \qquad if(it <= MAX\_IT/2) \\ 1/2 + rand()(1/2 - X_i^j(t)) \qquad else \end{array} \right. \end{aligned}$$

In feature selection, the positions of individuals are limited to [0,1], and then they are compared with a threshold of 0.5 to determine whether the corresponding feature is selected. At the beginning of the algorithm, Eq. (4) is the OBL (Opposition-based Learning), and individuals search in opposite directions. In the subsequent phase, individuals will randomly choose a search point at the current and opposite positions.

#### Repairing positions

In this study, the dimension of an individual is twice the number of emotional features (2 * *dim*), where *dim* represents the number of emotional features. The identification of male emotional features is based on 1 to *dim*, while female emotional features are represented by *dim*+1 to 2 * *dim*. Due to the physiological differences between males and females and the random nature of metaheuristic algorithms, their emotional features are not quite consistent. The individual’s position needs to be corrected, as demonstrated in Algorithm 1.

**Algorithm 1:**
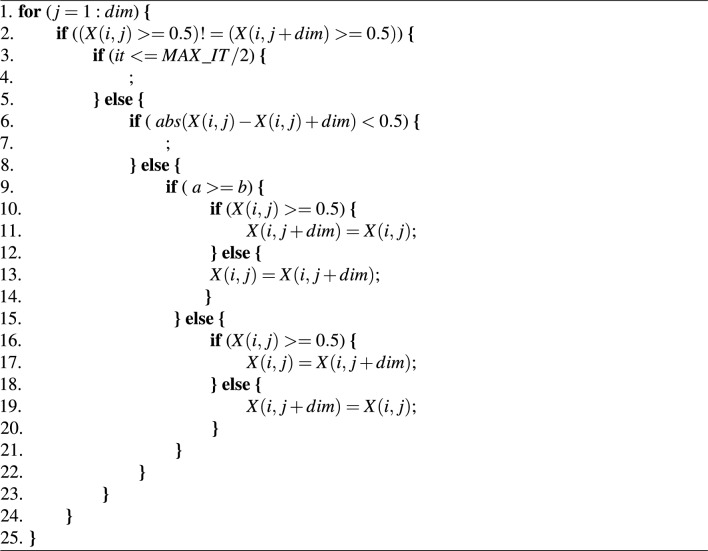
Repairing positions

Lines 3 and 8 imply that in the early stages or when the positions in male and female are similar, the algorithm won’t adjust the positions to enhance diversity among individuals. Lines 9-27 describe the correction process, where *a* and *b* represent the number of features selected for males and females in different historical optimal solutions achieved by the algorithm. Position correction ensures the consistency of acoustic features and also takes into account gender differences.

## Experimental results and discussions

We conduct experiments to verify the superiority of the proposed ADE algorithm against DE^37^, BBO_PSO^38^ and MA^29^ algorithms. BBO_PSO and MA are two state-of-the-art algorithms in emotion recognition. BBO_PSO focuses on emotion recognition, while MA classifies emotions based on gender. DE and ADE utilize the gender-emotion model shown in Fig. 1. Table 2 displays the main parameter settings of the compared algorithms.

**Table 2:** The main parameter settings.

Algorithms	Main parameters
BBO_PSO	pMutation = 0.1; KeepRate = 0.2;
MA	mu=0.01; nPop=10; nPopf=10; g=0.8; DANCE = 5;
DE	pCR = 0.2; F_min = 0.2; F_max = 0.8;
ADE	pCR = 0.2;

The algorithms are executed 20 times, and their population size is fixed at 20. DE, BBO_PSO, and MA have a maximum iteration of 100, while ADE has 200 iterations. To evaluate potential significant differences in the experimental results, we utilize the Wilcoxon rank sum test and the Friedman test, with a significance level set at 0.05.

### Objective function

Classification accuracy is the most important metric for SER algorithms, so it is utilized as the objective function in the experiments, as indicated in Eq. (5). Accuracy, weighted accuracy, and unweighted accuracy are metrics used to evaluate the classification ability of emotion recognition algorithms. Although weighted and unweighted accuracy better reflect a model’s performance in imbalanced categories, our emotion datasets contain multiple types, and they can test the performance of algorithms more comprehensively. Our objective is to maximize the recognition accuracy of these algorithms, which can be achieved through the evaluation of accuracy. We analyze the confusion matrix to identify which emotions the algorithm classifies well. Additionally, we evaluate the algorithms by comparing recall, precision, F1-Score, the number of selected features, and execution time.

5 $$\begin{aligned} accuracy = \dfrac{S1}{S1+S2} \end{aligned}$$

where *S*1 and *S*2 represent the numbers of correctly classified and incorrectly classified samples, respectively.

### Experimental analysis

We use SVM as the classifier, and we employ 10-fold cross-validation to assess the performance of the algorithms. For ease of reading, we mark the best experimental data obtained by the algorithms in bold font.

Table 3 displays the average, minimum, and maximum recognition accuracy. ADE exhibits superior classification accuracy compared to DE in CREMA-D, EmergencyCalls, and RAVDESS, whereas DE outperforms ADE only in IEMOCAP-S1. This suggests the effectiveness of the proposed DE improvement method. Additionally, ADE demonstrates better accuracy than BBO_PSO, MA, and DE in CREMA-D, EmergencyCalls, and RAVDESS, while DE has better average recognition than other algorithms in IEMOCAP-S1. The overall performance of the algorithms in IEMOCAP-S1 is general. The main reason for this is that the dataset contains the most emotional features and the sample distribution is uneven, which prevents the algorithms from creating accurate prediction models. In EmergencyCalls, ADE achieves the best prediction accuracy, and its worst prediction value is also better than BBO_PSO, MA, and DE. In IEMOCAP-S1, ADE attains the highest classification accuracy at 0.5729, outperforming other algorithms. Meanwhile, DE’s worst prediction value of 0.5578 is superior to that of other algorithms. In RAVDESS, ADE and DE outperform the comparison algorithms in the best and worst prediction values, respectively.

**Table 3:** The classification accuracies of the algorithms.

Datasets	BBO_PSO		MA		DE		ADE	
	AVG	Min/Max	AVG	Min/Max	AVG	Min/Max	AVG	Min/Max
CREMA-D	0.5715	0.5669/0.5771	0.6503	0.6470/0.6539	0.6568	0.6515/0.6629	**0.6617**	**0.6594**/**0.6642**
EmergencyCalls	0.6107	0.5768/0.6331	0.6036	0.5957/0.6139	0.6504	0.6426/0.6610	**0.6660**	**0.6543**/**0.6824**
IEMOCAP-S1	0.4702	0.4599/0.4787	0.5514	0.5461/0.5593	**0.5632**	**0.5578**/0.5693	0.5631	0.5526/**0.5729**
RAVDESS	0.7043	0.6868/0.7159	0.6896	0.6823/0.6982	0.7025	**0.6960**/0.7112	**0.7093**	0.6853/**0.7262**
Rank	3		3.75		2		1.25	
P-Value	0.0440							

Significant values are in [bold].

The Wilcoxon rank sum reveals that BBO_PSO has consistent statistical data with ADE in RAVDESS, and DE and ADE have similarities in IEMOCAP-S1. The average ranks of BBO_PSO, MA, DE, and ADE in CREMA-D, EmergencyCalls, IEMOCAP-S1, and RAVDESS are 3, 3.75, 2, and 1.25 respectively, with a P-Value of 0.0440. The Friedman test demonstrates that ADE performs best on emotional datasets. MA, DE and ADE are all gender-based emotion recognition algorithms, while BBO_PSO does not utilize gender to complete emotion recognition. From Table 3, we can see that the performance of DE and ADE is better than that of BBO_PSO. This indicates that gender information can improve emotion recognition accuracy.

To further validate the efficiency of the algorithms, we analyze the performance of them from precision, recall, and F1-score, as shown in Table 4. The algorithms are the most effective in RAVDESS, and the data is comparable in CREMA-D and EmergencyCalls. Since some emotional samples in IEMOCAP-S1 have less data, the algorithms cannot classify them correctly. Consequently, the data for precision and F1-score are unavailable, but they also have low recall values. ADE outperforms the comparison algorithms in precision, recall, and F1-score in CREMA-D, EmergencyCalls, and IEMOCAP-S1, but its performance in RAVDESS is surpassed by BBO_PSO. ADE outperforms BBO_PSO, MA, and DE in RAVDESS for classification accuracy, but lacks in recall and precision. The optimization goal of ADE is to improve overall classification accuracy rather than focus on the recognition ability for each class of samples. This may cause ADE to perform poorly in identifying rare or borderline samples, leading to missing some positive samples (lower recall) or misclassifying more negative samples (lower precision).

**Table 4:** The recall, precision, and F1-score of the algorithms.

Datasets	Algorithms	Recall	Precision	F1-score
CREMA-D	BBO_PSO	0.5735	0.5718	0.5696
EmergencyCalls		0.6152	0.6276	0.6201
IEMOCAP-S1		0.2658	NaN	NaN
RAVDESS		**0.7017**	0.7420	0.6822
CREMA-D	MA	0.6493	0.6497	0.6459
EmergencyCalls		0.6010	0.6581	0.5527
IEMOCAP-S1		0.3206	NaN	NaN
RAVDESS		0.6867	0.7324	0.6617
CREMA-D	DE	0.6559	0.6570	0.6530
EmergencyCalls		0.6535	0.6896	0.6128
IEMOCAP-S1		**0.3338**	NaN	NaN
RAVDESS		0.7013	**0.7441**	0.6789
CREMA-D	ADE	**0.6606**	**0.6629**	**0.6577**
EmergencyCalls		**0.6710**	**0.7056**	**0.6318**
IEMOCAP-S1		0.3318	NaN	NaN
RAVDESS		0.6964	0.7148	**0.6978**

Significant values are in [bold].

Table 5 presents the running time and the number of selected features. BBO_PSO has a clear advantage in running time, and it has better operational efficiency than MA, DE, and ADE in EmergencyCalls, IEMOCAP-S1, and RAVDESS. ADE achieves the shortest running time in CREMA-D. The time complexity of the SVM classifier is between O($D * T^2$) and O($D * T^3$), where *D* means the feature size and *T* implies the number of samples. The calculation time of feature selection algorithms is mainly affected by classifiers. Although BBO_PSO employs more features than ADE, the time difference between them is marginal. ADE takes less time than DE on the four datasets. Because CREMA-D contains the largest number of samples and EmergencyCalls has the smallest sample size, the calculation time of the algorithms in CREMA-D is considerably longer than in the other datasets.

The algorithms obtain the same number of features from the datasets, so the numbers of features they selected in each dataset are similar. ADE utilizes fewer features compared to the other algorithms. On the other hand, DE employs the most features, but it’s important to note that both ADE and DE explore twice the feature space of BBO_PSO and MA.

**Table 5:** The number of selected features and running time of the algorithms.

Datasets	BBO_PSO		MA		DE		ADE	
	Length	Time	Length	Time	Length	Time	Length	Time
CREMA-D	66.85	30932.10	74.55	20270.67	130.20	19478.65	**80.00**	**19032.58**
EmergencyCalls	54.55	**653.00**	72.10	1194.03	108.75	1203.82	**49.70**	1202.07
IEMOCAP-S1	57.20	**6554.87**	72.95	6920.19	113.40	7151.13	**52.25**	7023.83
RAVDESS	56.70	**4290.22**	71.95	5485.30	113.75	5328.26	**53.15**	5302.73

Significant values are in [bold].

### Discussion

The time complexity of ADE is $$O(G*N*dim+G*N*f)$$, where *f* is the execution time of the objective function, and *G* and *N* represent the maximum iteration and population size. In feature selection, due to the high complexity of *f*, the maximum time complexity can also be represented as $$O(G*N*f)$$.

**Figure 3:**
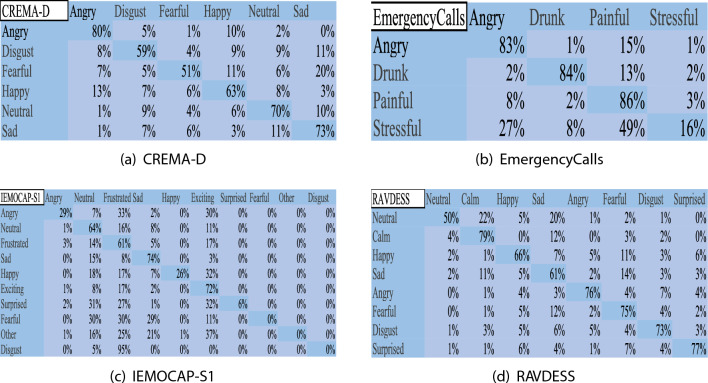
The confusion matrix of ADE.

Figure 3 depicts the confusion matrix of ADE. In CREMA-D, ADE recognizes Angry, Neutral, and Sad well, but in Disgust and Fearful, Sad, Happy, and Angry greatly interfere with the accuracy. In EmergencyCalls, the recognition of Angry, Drunk, and Stressful is affected by the presence of Painful. ADE performs remarkably well in identifying Painful, Angry, and Drunk, but it’s hard to distinguish Stressful. In IEMOCAP-S1, the number of emotion samples for Surprised, Fearful, Other, and Distinct is relatively small. It is difficult for ADE to make correct judgments, and they do not affect the recognition of other emotions. The algorithm’s emotion recognition is complicated by Neutral and Frustrated emotions, and ADE has the best accuracy in classifying Sad and Exciting emotions. In RAVDESS, ADE is the top performer in recognizing Calm, Angry, Fearful, Disgust, and Surprised. However, the algorithm could easily mistake Neutral for Calm and Sad.

In terms of acoustic features, males and females have distinct differences in the following aspects:

1. The mean of MFCC [3,4,6,7,8,14] and log Mel Freq. Band [6,7] in CREMA-D.
2. The mean and differentiation of MFCC [4,6,7,8,13,14], log Mel Freq. Band [0,7], and LSP Frequency [1] in EmergencyCalls.
3. The mean, variance, and second-order differentiation of MFCC [0,1,4,9,10,11,12], LSP Frequency [0], and PCM Loudness in IEMOCAP-S1.
4. The mean, variance, and differentiation of MFCC [3,6,7,8,9,13], F0 Envelope Quartile, and F0 by Sub Hungarian Sum in RAVDESS.

Gender information in MFCC^3,6–9,13^ is significantly different, and the mean, variance, and differentiation of features also have statistical characteristics that impact recognition.

Recently, speech emotions have been recognized through CNN and feature fusion^39,40^.^39^ claimed an accuracy of 58.62% in RAVDESS, and it can be increased to 78.35% using data augmentation. By combining the frequency and time-domain features of MFCC^40^, reported that the recognition rates of IEMOCAP and RAVDESS can be improved to 74.62% and 86.11% using the obtained 23712 features. In the future, we can utilize data augmentation and feature fusion methods to improve the classification performance of the ADE algorithm.

## Conclusions

The proposed emotion recognition system comprises two parts: 1) gender recognition, and 2) emotion recognition, both of which use the SVM classifier to complete predictions. A variety of features are extracted from speech signals, and feature selection is employed to establish the emotion recognition model. The recognition accuracies of ADE reach 0.6617, 0.6660, 0.5631, and 0.7093 on four datasets, which outperforms BBO_PSO, MA, and DE. It also exhibits strong performance in precision, recall, and F1-score. When recognizing speech features, it is important to take into consideration gender differences between males and females. ADE uses minimal features and still achieves satisfactory recognition while reducing running time. Through the confusion matrix, it is found that the algorithm’s recognition of emotions varies on different datasets. Moreover, the emotions that affect gender differences also vary with datasets, which poses challenges for real-world applications of the algorithm. In future work, we will analyze speech factors and propose corresponding models based on different scenarios.

## Data Availability

Data is available from the corresponding author on reasonable request, and this paper does not involve humans.

## References

[1] Zhao, Y. & Shu, X. Speech emotion analysis using convolutional neural network (cnn) and gamma classifier-based error correcting output codes (ecoc). *Sci. Rep.* **13**, 20398 (2023). 37989782 10.1038/s41598-023-47118-4 PMC10663497

[2] Arias Sarah, P. *et al.* Pupil dilation reflects the dynamic integration of audiovisual emotional speech. *Sci. Rep.* **13**, 5507 (2023). 37016041 10.1038/s41598-023-32133-2 PMC10073148

[3] Zhang, B., Provost, E. M. & Essl, G. Cross-corpus acoustic emotion recognition from singing and speaking: A multi-task learning approach. In *2016 IEEE International Conference on Acoustics, Speech and Signal Processing (ICASSP)*, 5805–5809 (IEEE, 2016).

[4] Lausen, A. & Schacht, A. Gender differences in the recognition of vocal emotions. *Front. Psychol.* **9**, 882 (2018). 29922202 10.3389/fpsyg.2018.00882 PMC5996252

[5] Akinpelu, S. & Viriri, S. Speech emotion classification using attention based network and regularized feature selection. *Sci. Rep.* **13**, 11990 (2023). 37491423 10.1038/s41598-023-38868-2 PMC10368662

[6] Shekhar, S. *et al.* Hemodynamic responses to emotional speech in two-month-old infants imaged using diffuse optical tomography. *Sci. Rep.* **9**, 4745 (2019). 30894569 10.1038/s41598-019-39993-7 PMC6426868

[7] Zhao, H. & Wang, P. A short review of age and gender recognition based on speech. In *2019 IEEE 5th Intl Conference on Big Data Security on Cloud (BigDataSecurity), IEEE Intl Conference on High Performance and Smart Computing,(HPSC) and IEEE Intl Conference on Intelligent Data and Security (IDS)*, 183–185 (IEEE, 2019).

[8] Kaur, H. & Kadyan, V. Feature space discriminatively trained punjabi children speech recognition system using kaldi toolkit. In *Proceedings of the International Conference on Innovative Computing & Communications (ICICC)* (2020).

[9] Singh, A., Kaur, N., Kukreja, V., Kadyan, V. & Kumar, M. Computational intelligence in processing of speech acoustics: A survey. *Complex Intell. Syst.* **8**, 2623–2661 (2022). 10.1007/s40747-022-00665-1

[10] Kappen, M. *et al.* Acoustic speech features in social comparison: How stress impacts the way you sound. *Sci. Rep.* **12**, 22022 (2022). 36539505 10.1038/s41598-022-26375-9 PMC9767914

[11] Hasija, T., Kadyan, V. & Guleria, K. Out domain data augmentation on punjabi children speech recognition using tacotron. In *Journal of Physics: Conference Series*, vol. 1950, 012044 (IOP Publishing, 2021).

[12] Zhu, Y., Li, W. & Li, T. A hybrid artificial immune optimization for high-dimensional feature selection. *Knowl.-Based Syst.* **260**, 110111 (2023). 10.1016/j.knosys.2022.110111

[13] Zhang, L.-M. *et al.* A deep learning method using gender-specific features for emotion recognition. *Sensors* **23**, 1355 (2023). 36772395 10.3390/s23031355 PMC9921859

[14] Hu, P., Pan, J.-S., Chu, S.-C. & Sun, C. Multi-surrogate assisted binary particle swarm optimization algorithm and its application for feature selection. *Appl. Soft Comput.* **121**, 108736 (2022). 10.1016/j.asoc.2022.108736

[15] Pan, J.-S., Song, P.-C., Chou, J.-H., Watada, J. & Chu, S.-C. Fpga-based compact differential evolution for general-purpose optimization in resource-constrained devices. *IEEE Trans. Ind. Inform.* (2024).

[16] Pan, J.-S., Hu, P., Snášel, V. & Chu, S.-C. A survey on binary metaheuristic algorithms and their engineering applications. *Artif. Intell. Rev.* **56**, 6101–6167 (2023). 36466763 10.1007/s10462-022-10328-9 PMC9684803

[17] Zeng, Z., Zhang, M., Chen, T. & Hong, Z. A new selection operator for differential evolution algorithm. *Knowl.-Based Syst.* **226**, 107150 (2021). 10.1016/j.knosys.2021.107150

[18] Gupta, S. & Su, R. Multiple individual guided differential evolution with time varying and feedback information-based control parameters. *Knowl.-Based Syst.* **259**, 110091 (2023). 10.1016/j.knosys.2022.110091

[19] Lin, M., Wang, Z., Chen, D. & Zheng, W. Particle swarm-differential evolution algorithm with multiple random mutation. *Appl. Soft Comput.* **120**, 108640 (2022). 10.1016/j.asoc.2022.108640

[20] Wang, X., Wang, Y., Wong, K.-C. & Li, X. A self-adaptive weighted differential evolution approach for large-scale feature selection. *Knowl.-Based Syst.* **235**, 107633 (2022). 10.1016/j.knosys.2021.107633

[21] Hasija, T. *et al.* Prosodic feature-based discriminatively trained low resource speech recognition system. *Sustainability* **14**, 614 (2022). 10.3390/su14020614

[22] Kadyan, V., Mantri, A. & Aggarwal, R. Improved filter bank on multitaper framework for robust punjabi-asr system. *Int. J. Speech Technol.* **23**, 87–100 (2020). 10.1007/s10772-019-09654-1

[23] Zhang, B., Provost, E. M. & Essl, G. Cross-corpus acoustic emotion recognition with multi-task learning: Seeking common ground while preserving differences. *IEEE Trans. Affect. Comput.* **10**, 85–99 (2017). 10.1109/TAFFC.2017.2684799

[24] Sun, T.-W. End-to-end speech emotion recognition with gender information. *IEEE Access* **8**, 152423–152438 (2020). 10.1109/ACCESS.2020.3017462

[25] Velichko, A. *et al.* Complex paralinguistic analysis of speech: Predicting gender, emotions and deception in a hierarchical framework. *INTERSPEECH* **2022**, 4735–4739 (2022). 10.21437/Interspeech.2022-11294

[26] Aggarwal, G. & Vig, R. Acoustic methodologies for classifying gender and emotions using machine learning algorithms. In *2019 Amity International Conference on Artificial Intelligence (AICAI)*, 672–677 (IEEE, 2019).

[27] Mishra, P. & Sharma, R. Gender differentiated convolutional neural networks for speech emotion recognition. In *2020 12th International Congress on Ultra Modern Telecommunications and Control Systems and Workshops (ICUMT)*, 142–148 (IEEE, 2020).

[28] Latif, S. *et al.* Multi-task semi-supervised adversarial autoencoding for speech emotion recognition. *IEEE Trans. Affect. Comput.* **13**, 992–1004 (2020). 10.1109/TAFFC.2020.2983669

[29] Garain, A. *et al.* Grann: feature selection with golden ratio-aided neural network for emotion, gender and speaker identification from voice signals. *Neural Comput. Appl.* **34**, 14463–14486 (2022). 10.1007/s00521-022-07261-x

[30] Yao, Z., Wang, Z., Liu, W., Liu, Y. & Pan, J. Speech emotion recognition using fusion of three multi-task learning-based classifiers: Hsf-dnn, ms-cnn and lld-rnn. *Speech Commun.* **120**, 11–19 (2020). 10.1016/j.specom.2020.03.005

[31] Liu, Y., Sun, H., Guan, W., Xia, Y. & Zhao, Z. Speech emotion recognition using cascaded attention network with joint loss for discrimination of confusions. *Mach. Intell. Res.* **20**, 595–604 (2023). 10.1007/s11633-022-1356-x

[32] Cao, H. *et al.* Crema-d: Crowd-sourced emotional multimodal actors dataset. *IEEE Trans. Affect. Comput.* **5**, 377–390 (2014). 25653738 10.1109/TAFFC.2014.2336244 PMC4313618

[33] Lichman, M. uci machine learning repository (Available at: (http://archive.ics.uci.edu/ml. Irvine, CA: University of California, School of Information and Computer Science ))(2013).

[34] Jia, N., Zheng, C. & Sun, W. A multimodal emotion recognition model integrating speech, video and mocap. *Multimedia Tools Appl.* **81**, 32265–32286 (2022). 10.1007/s11042-022-13091-9

[35] Livingstone, S. R. & Russo, F. A. The ryerson audio-visual database of emotional speech and song (ravdess): A dynamic, multimodal set of facial and vocal expressions in north american english. *PLoS ONE* **13**, e0196391 (2018). 29768426 10.1371/journal.pone.0196391 PMC5955500

[36] Schuller, B. *et al.* The interspeech 2010 paralinguistic challenge. In *Proc. INTERSPEECH 2010, Makuhari, Japan*, 2794–2797 (2010).

[37] Gharsellaoui, S., Selouani, S.-A. & Yakoub, M. S. Linear discriminant differential evolution for feature selection in emotional speech recognition. In *INTERSPEECH*, 3297–3301 (2019).

[38] Yogesh, C. *et al.* Hybrid bbo_pso and higher order spectral features for emotion and stress recognition from natural speech. *Appl. Soft Comput.* **56**, 217–232 (2017). 10.1016/j.asoc.2017.03.013

[39] Namey, A. & Akter, K. Cochleation: Speech emotion recognition through cochleagram with cnn-gru and attention mechanism. In *2024 6th International Conference on Electrical Engineering and Information & Communication Technology (ICEEICT)*, 01–06 (IEEE, 2024).

[40] Zhang, P. *et al.* Lightweight fusion model with time-frequency features for speech emotion recognition. In *2024 27th International Conference on Computer Supported Cooperative Work in Design (CSCWD)*, 3017–3022 (IEEE, 2024).

